# Prognostic factors for local control of stage I non-small cell lung cancer in stereotactic radiotherapy: a retrospective analysis

**DOI:** 10.1186/1748-717X-7-182

**Published:** 2012-10-31

**Authors:** Yuko Shirata, Keiichi Jingu, Masashi Koto, Masaki Kubozono, Ken Takeda, Toshiyuki Sugawara, Noriyuki Kadoya, Haruo Matsushita

**Affiliations:** 1 Department of Radiation Oncology, Tohoku University School of Medicine, Sendai, Japan; 2 Research Center for Charged Particle Therapy, National Institute of Radiological Sciences, Chiba, Japan; 3 Department of Therapeutic Radiology, Clinical Radiological Science, Course of Radiological Technology, Tohoku University School of Health Sciences, Sendai, Japan; 4 Department of Radiation Oncology, Tohoku University School of Medicine, 1-1 Seiryo-chou, Aoba-ku, Sendai 980-8574, Japan

**Keywords:** Stereotactic radiotherapy, SBRT, Non-small cell lung cancer, NSCLC, Prognostic factor, Minimum dose, PTV margin

## Abstract

**Background:**

The purpose of this study is to investigate the prognostic factors of stereotactic radiotherapy for stage I NSCLC to improve outcomes.

**Methods:**

Stage I non-small cell lung cancer patients who were treated with stereotactic radiotherapy between 2005 and 2009 at our hospital were enrolled in this study. The primary endpoint was local control rate. Survival estimates were calculated from the completion date of radiotherapy using the Kaplan-Meier method. The prognostic factors including patients’ characteristics and dose-volume histogram parameters were evaluated using Cox’s proportional hazard regression model.

**Results:**

Eighty patients (81 lesions) treated with 3 dose levels, 48 Gy/4 fractions, 60 Gy/8 fractions and 60 Gy/15 fractions, were enrolled in this study. Median follow-up was 30.4 months (range, 0.3 – 78.5 months). A Cox regression model showed T factor (*p* = 0.013), biological effective dose calculated from prescribed dose (BED_10_) (*p* = 0.048), and minimum dose for PTV (*p* = 0.013) to be prognostic factors for local control. Three-year overall survival rate and local control rate were 89.9% (T1: 86.8%, T2: 100%) and 89.0% (T1: 97.9%; T2: 64.8%), respectively. When the 3-year local control rates were examined by prescribed doses, they were 100% for the dose per fraction of 48 Gy /4 fractions (105.6 Gy BED_10_), 82.1% for 60 Gy/8 fractions (105 Gy BED_10_), and 57.1% for 60 Gy/15 fractions (84 Gy BED_10_). The median value of the minimum dose for PTV (%) was 89.88 (%), and the 3-year local control rates were 100% in those with the minimum dose for PTV (%) ≥ 89.88% and 79.2% in those with the minimum dose for PTV (%) < 89.88%.

**Conclusions:**

Our results suggest that T factor, BED_10_, and minimum dose for PTV influence the local control rate. Local control rate can be improved by securing the minimum dose for PTV.

## Background

In stereotactic radiotherapy for lung tumors, the dose at the lesion has been successfully increased through advancement of irradiation devices, improvement of set-up accuracy, introduction of image-guidance technology and measures for respiratory tumor movement [1,2] while ensuring a high level of safety. For stage I non-small cell lung cancer (NSCLC), in particular, some reports suggest that short-term outcomes of stereotactic radiotherapy are comparable to those of surgeries [3,4]. Furthermore, patients diagnosed with lung cancer in the early stage have increased recently due to the use of computed tomography (CT) scans and educational campaigns for cancer screening [5,6].

However, lung cancer is still the main cause of cancer death worldwide, and local recurrence after stereotactic radiotherapy is not rare [3,7-9]. The purpose of this study is to investigate the prognostic factors of stereotactic radiotherapy for stage I NSCLC to improve outcomes.

## Methods

### Patient eligibility

We reviewed data for all of the 80 patients (81 lesions) with stage I NSCLC who had undergone stereotactic radiotherapy in our hospital between March 2005 and July 2009. All patients included in this study had histological or cytological diagnosis of NSCLC and were staged as Union Internationale Contre le Cancer (UICC)-6 stage IA or IB by the use of CT. If available, 18 F-fluorodeoxyglucose positron emission tomography (FDG-PET) was used for staging. They either had a medical contraindication to surgery or refused surgery. Patient eligibility was not restricted on the basis of tumor location, unless a part of the esophagus, heart, main bronchi, hilus, spinal cord, or skin would be exposed to high-dose radiation. If the treatment plan included these organs in the high-dose areas, the patients were treated with conventional radiotherapy or modified stereotactic radiotherapy with a moderate irradiation dose; those patients were excluded from this study.

The primary endpoint was local recurrence. The secondary endpoint was overall survival. Patients’ background factors, various clinical parameters, and clinical course after irradiation were surveyed using information sources including medical records, data saved at the practice support terminal of our hospital, case follow-up cards of our department, and irradiation records. Local recurrence was defined as local progression that was 1.5 times or more the dimensions of original tumor [10]. Tumors were observed on CT and/or 18 F-fluorodeoxyglucose positron emission tomography (FDG-PET) in order to assess the primary tumor’s stage and the presence or absence of recurrence. If FDG-PET was available, the maximum standardized uptake value (SUVmax) greater than 5.0 was considered as recurrence [11]. The physicians and radiation oncologists finally decided to be local recurrence.

The study protocol was approved by the Ethical Committee of our institution, and informed consent was obtained from all patients.

### Radiotherapy

Treatment factor is summarized in Table 1. To determine the extent of tumor movement due to respiration and establish an individual internal margin, all patients were placed in a simulator for fluoroscopic examination just prior to CT scanning for treatment planning in the exact same position. Serial CT scans were performed at intervals of 2.5 mm. A CT scan with an acquisition time of 4 seconds that included internal motion was performed to define the internal margin accurately.

**Table 1:** Treatment factor

**Computed tomography**	- Long time scan
	- 2.5 mm slice
**Radiotherapy planning system**	- Eclipse
	-Algorithm: Pencil beam convolution
	- Heterogeneity correction: Modified Batho Power Law
**Targeting**	- Observation of the tumor by fluoroscopy in advance
	- GTV + 0–5 mm = CTV, CTV + 5–10 mm = PTV
**Irradiation field**	- non-coplanar multi-dynamic arcs and/or multi-static beams
**Prescription**	- 48 Gy/4 fractions, 60 Gy/8 fractions, 60 Gy/15 fractions
	- Prescription for the iso-center
	- 6 MV - X ray
**Radiation therapy equipment**	- Clinac 23EX

Patients were immobilized in the supine position with an individually fashioned half-body vacuum cast. Both the upper extremities were immobilized in the raised position unless the tumor was located at the apex of the lung, in which case both the upper extremities were immobilized beside the body.

Gross tumor volume (GTV) was defined as the visible extent of the tumor on the CT image at the lung window. Clinical target volume (CTV) was defined as GTV plus 0–5 mm margin for microscopic invasion. Internal target volume (ITV) was set equal to CTV because CT scanning was performed with an acquisition time of 4 seconds, and we consider that long-time (4 seconds) scan CT depicted virtually the entire tumor trajectory [12]. Planning target volume (PTV) was determined by allowing for a set-up margin of 5 - 10mm beyond the ITV.

Treatment planning was performed with non-coplanar multi-dynamic arcs and/or multi-static beams by using a three-dimensional radiotherapy treatment planning system (Eclipse, Varian Medical Systems, Palo Alto, CA). The algorithm to calculate the dose was pencil beam convolution (PBC). Modified Batho Power law correction was used as the tissue heterogeneity correction algorithm. The target reference point was defined as the center of the PTV, and the dose was prescribed for its point. PTV was encompassed by the minimum 90% dose line of the reference point dose as possible. X-rays of 6 MV were used in all treatments.

The treatment took place using Clinac 23EX, Varian Medical Systems, Palo Alto, CA. In each irradiation, the position of the tumor was confirmed with fluoroscopy, and set-up and/or inter-fractional errors were corrected.

The patients were treated with a radiation schedule of 12 Gy /fraction ×4 fractions (48 Gy/4 fractions), 7.5 Gy/fraction ×8 fractions (60 Gy/8 fractions), and 4 Gy/fraction ×15 fractions (60 Gy/15 fractions). When the tumor was close to a risk organ, 7.5 Gy/fraction ×8 fractions or 4 Gy/fraction ×15 fractions was used to reduce the risk of serious toxicity due to set-up error or internal motion.

### Follow-up

The first examination, including a clinical examination and CT scanning, was performed 4–6 weeks after treatment to assess the pulmonary reaction. Thereafter, the patients underwent follow-up examinations every 3–6 months for 2 years following treatment. After 2 years, follow-up examinations were performed every 6 months.

### Statistical analysis

The prognostic factors for local control, including age, sex, T factor, histology, planning target volume (PTV), minimum dose for PTV (%), biological effective dose (BED) calculated from prescribed dose (BED_10_), and biological effective dose calculated from minimum dose (BEDmin), were investigated by stepwise Cox’s proportional hazard regression model for multivariate analysis. Hazard ratio for continuous data was evaluated with Wald χ^2^ test statistics. Sex and T factor were considered as categorical data. To investigate for the presence of multicollinearity, correlation coefficients were calculated for all variables. The hazard ratio was observed graphically to check its constancy.

BED_10_ and BEDmin were calculated using the linear quadratic formula in order to compare the effects of treatments with different fraction sizes and total doses. BED was given by: BED= nd[1 + d/(*α*/*β*)], where n is the number of fractions, d is the dose/fraction, and *α*/*β* ratio is 10 Gy. BED was not corrected with values for overall radiation time or tumor doubling time.

Overall survival, cause specific, and local control rates were calculated using the Kaplan-Meier method and statistical differences were evaluated by the log-rank test. When a continuous data was used as a variable for the Kaplan-Meier method, the data was divided by the median value into two groups. Statistical significance was defined as a value of p<0.05 in the present study. All analyses were performed using the SPSS 17.0 software package (SPSS Inc, Chicago, IL).

## Results

### Patients

All eighty patients were enrolled in the present study. The patients’ characteristics are shown in Table 2. There were 64 men (64 lesions) and 16 women (17 lesions) with ages ranging from 54 to 90 years (median, 70 years). All of the patients completed the treatment without acute adverse effects. The observation periods from the time of completion of stereotactic radiotherapy ranged from 0.3 to 78.5 months with a median of 30.4 months. The patients’ histologies were adenocarcinoma (33 patients), squamous cell carcinoma (22 patients), large cell carcinoma (5 patients), and unclassified NSCLC (20 patients). Sixty-three tumors were T1 (Stage IA) masses and 18 tumors were T2 (Stage IB). Performance status (PS) of the patient was 0–2.

**Table 2:** Patient backgrounds

**Age**	median, 77 years (range: 54–90 years)
**Sex**	Male: 64 lesions, 64 patients
	Female: 17 lesions, 16 patients
**Histopathology**	Adenocarcinoma: 33 patients
	Squamous cell carcinoma: 22 patients
	Large cell carcinoma: 5 patients
	Unclassified: 20 patients
**Stage**	T1 (Stage IA): 63 lesions
	T2 (Stage IB): 18 lesions

Fifteen (18.8%) of the 80 patients showed evidence of recurrence. Local, regional (nodal) and distant recurrences were observed in 6 patients (7.5%), 3 patients (3.8%) and 12 patients (15%), respectively. Time to local failure varied between 12.2 and 33.7 months (median, 18.1 months).

Four patients died of NSCLC treated with stereotactic radiotherapy and 6 patients died of intercurrent causes. The 4 patients who died of NSCLC treated with stereotactic radiotherapy included 1 patient with local disease and regional lymph node metastasis and 3 patients with distant metastases. Intercurrent causes were colorectal cancer, aspiration pneumonia, advanced esophageal cancer, renal failure, chronic obstructive pulmonary disease (COPD), and multiple liver metastases considered to be from another site of NSCLC.

### Radiotherapy

We treated 45 patients with a radiation schedule of 12 Gy /fraction ×4 fractions, 29 patients with a schedule of 7.5 Gy/fraction ×8 fractions and 7 patients with a schedule of 4 Gy/fraction ×15 fractions. We treated the tumor in consecutive weekdays.

### Statistical analysis

The results of multivariate analysis showed significant differences in T factor, BED_10_ and minimum dose for PTV (%). The hazard ratios were 0.027, 0.383 and 0.731, respectively (Table 3). Namely, the hazard ratio of T1 was 0.027 on defining the hazard ratio of T2 as 1.0. In minimum dose for PTV, the hazard ratio for each 1% increase was 0.731 for local recurrence. In BED_10_ (Gy), the hazard ratio for each 10 Gy increase was 0.383. Wald χ^2^ test indicated that minimum dose for PTV (%) was the strongest prognostic factor among these variables. There was no multicollinearity problem in this study. We observed constant hazard ratio graphically.

**Table 3:** Multivariate analysis with variables selected by stepwise method

**Variable [Reference]**	**Hazard ratio**	**χ**^**2**^	**95% confidence interval of hazard ratio**	**Significance probability (*p*)**
**T factor [Group: T2]**	0.027	6.142	0.002 – 0.470	0.013
**Minimum dose for PTV (%) [Unit: 1%]**	0.731	6.203	0.571 – 0.935	0.013
**BED**_**10**_**(Gy) [Unit: 10 Gy]**	0.383	3.898	0.148 – 0.993	0.048

**Figure 1:**
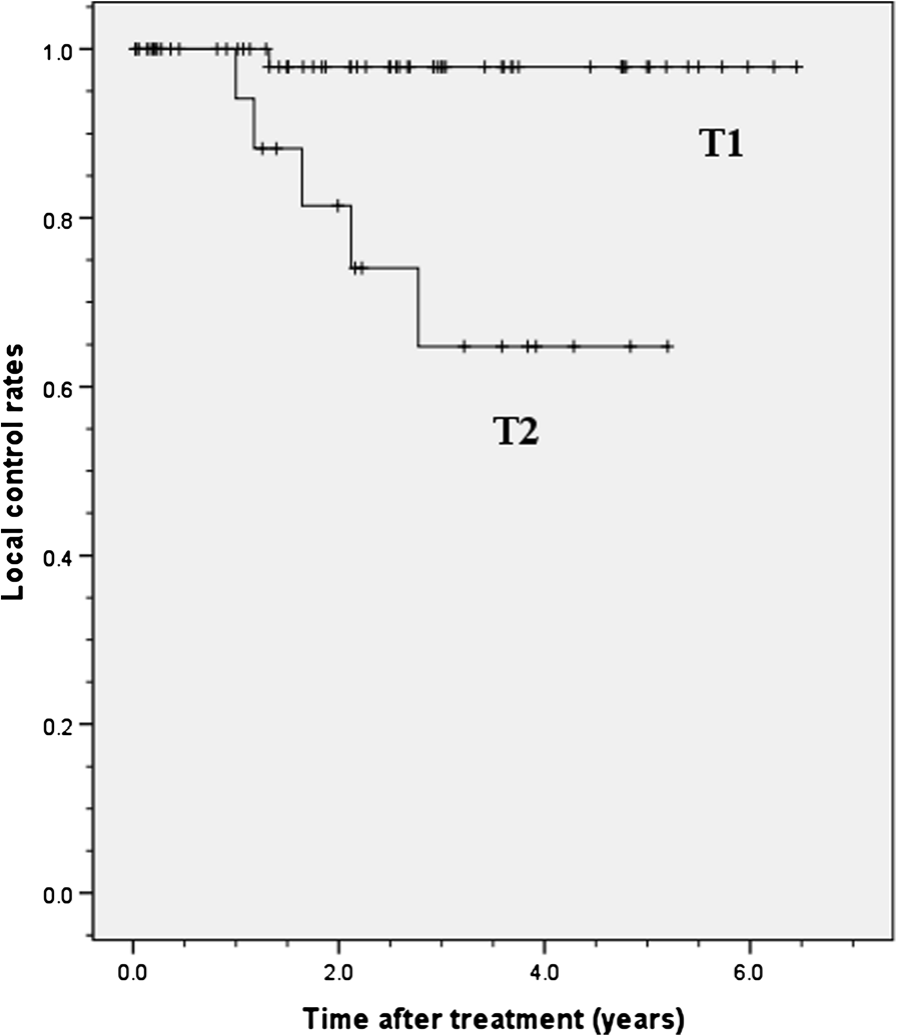
**Local control rates of T1 and T2 tumors treated with stereotactic radiotherapy.** The 3-year local control rates were 97.9% in patients with T1 tumors and 64.8% in those with T2 tumors.

**Figure 2:**
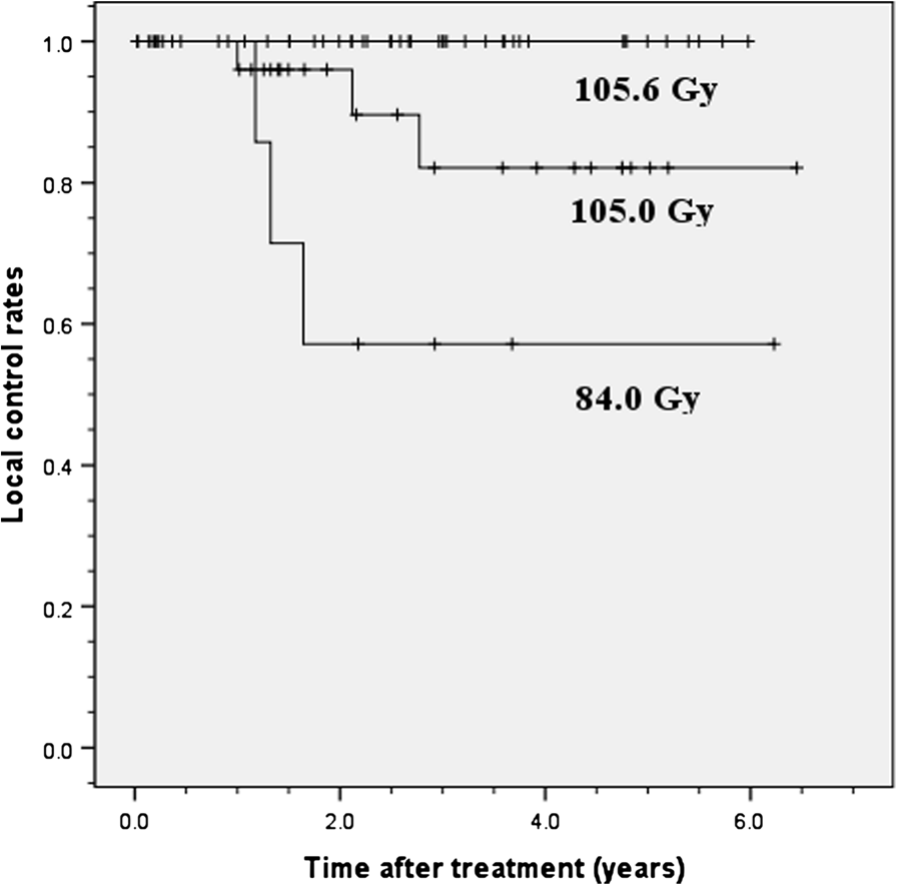
**Local control rate by BED**_**10**_**.** The 3-year local control rates were 100% in treatment with 105.6 Gy BED_10_ (48 Gy/4 fractions), 82.1% in treatment with 105.0 Gy BED_10_ (60 Gy/8 fractions) and 57.1% in treatment with 84.0 Gy BED_10_ (60 Gy/15 fractions).

**Figure 3:**
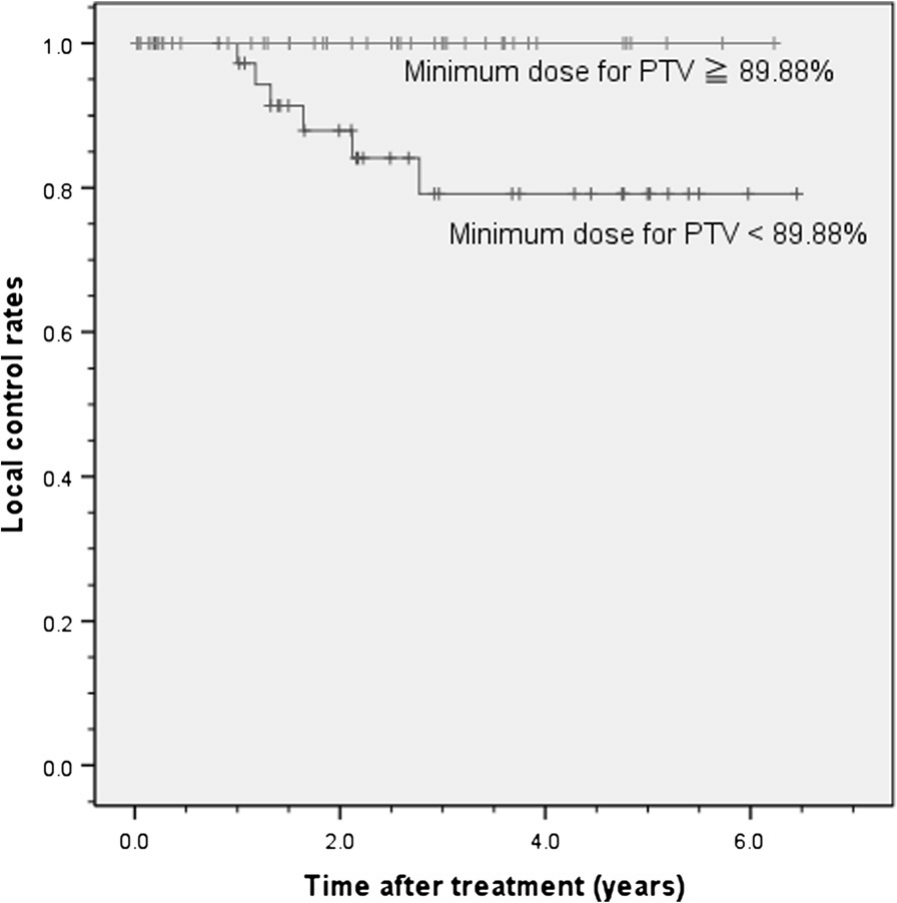
**Local control rate by the minimum dose for PTV (%).** The 3-year local control rates were 100% in those with the minimum dose for PTV (%) ≥ 89.88% and 79.2% (95% CI, 63.9% – 94.5%) in those with the minimum dose for PTV (%) < 89.88%. 89.88% is the median value of the minimum dose for PTV (%).

The 3-year local control rates were 89.0% (95% confidence interval [CI], 80.4% – 97.6%) in all patients, 97.9% (95% CI, 93.8% – 102.0%) in those with T1 tumors and 64.8% (95% CI, 38.9% – 90.7%) in those with T2 tumors. The log-rank test showed a significant difference between these two groups (*p* = 0.001, Figure 1). When the 3-year local control rates were examined by prescribed doses, they were 100% for the dose per fraction of 12 Gy /fraction ×4 fractions (105.6 Gy BED_10_), 82.1% (95% CI, 63.1% – 101.1%) for 7.5 Gy/fraction ×8 fractions (105 Gy BED_10_), and 57.1% (95% CI, 20.4% – 93.8%) for 4 Gy/fraction ×15 fractions (84 Gy BED_10_) with a significant difference (*p* = 0.001, Figure 2). The median value of the minimum dose for PTV (%) was 89.88 (%), and the 3-year local control rates were 100% in those with the minimum dose for PTV (%) ≥ 89.88% and 79.2% (95% CI, 63.9% – 94.5%) in those with the minimum dose for PTV (%) < 89.88%. The log-rank test showed a significant difference between these two groups (*p* = 0.016, Figure 3).

The 3-year overall survival rate for all patients was 89.9% (95% CI, 81.9% – 97.9%). The 3-year overall survival rates were 86.8% (95% CI, 76.6% – 97.0%) in patients with T1 tumors and 100% in those with T2 tumors. No significant difference was observed between these two groups (log-rank test, *p* = 0.29). The 3-year cause specific survival rate was 97.0% (95% confidence interval, 92.9% - 101.1%).

## Discussion

Multivariate analysis indicated that T factor, BED_10_, and minimum dose for PTV (%) influence local control. Among these, BED and minimum dose for PTV (%) can be changed by artificial intervention. BED_10_ would depend on the dose per fraction and total dose at a dose prescription, and they are generally determined by patient’s factor including tumor location, size, and patient’s general condition. The minimum dose for PTV (%) is decided by the radiotherapy plan including a targeting, margin factor, respiratory gating technique, and tumor tracking method. The results of this study suggest that the local control rate after stereotactic radiotherapy for stage I NSCLC can be improved by securing the minimum dose for PTV (%) when radiation oncologists produce a radiotherapy plan.

### Prognostic factors

Onimaru *et al*. analyzed 41 patients with stage I NSCLC treated by stereotactic radiotherapy and reported that T factor and prescribed dose were significant factors for local control in multivariate analysis. The margin status was not a significant factor in spite of the fact that it was used as a variable [2]. Probable reasons were the heterogeneity of the patients and the confounding factors analyzed in their study. Patients in whom a narrow margin was used included 7 patients with a T1 tumor and 3 patients with a T2 tumor.

In contrast, another study showed that tumor diameter and gender were the most significant factors affecting outcomes after stereotactic radiotherapy in recursive partitioning analysis; for comparison, tumor diameter was the only significant factor for local progression in a Cox proportional hazards model, and no margin-related variable was used [13]. This was possibly because of the female pathological inclination to have adenocarcinoma. Some reports suggest that adenocarcinoma patients with stage I NSCLC tend to have a better survival [14]. Since only 16 (20%) of the patients in our study were females, it was difficult to determine whether female gender is a significant prognostic factor.

Small sample size and retrospective protocol limit further interpretation of such findings, while tumor size is regarded as an independent prognostic factor for stage I NSCLC patients in general [14]. Multi-institutional phase II trials are currently be conducted by the Radiation Therapy Oncology Group (Protocol 0236) and Japan Clinical Oncology Group (Protocol 0403). Results of detailed statistical analysis of data obtained in these trials are awaited to clarify various issues including issues discussed below.

### T factor

**Figure 4:**
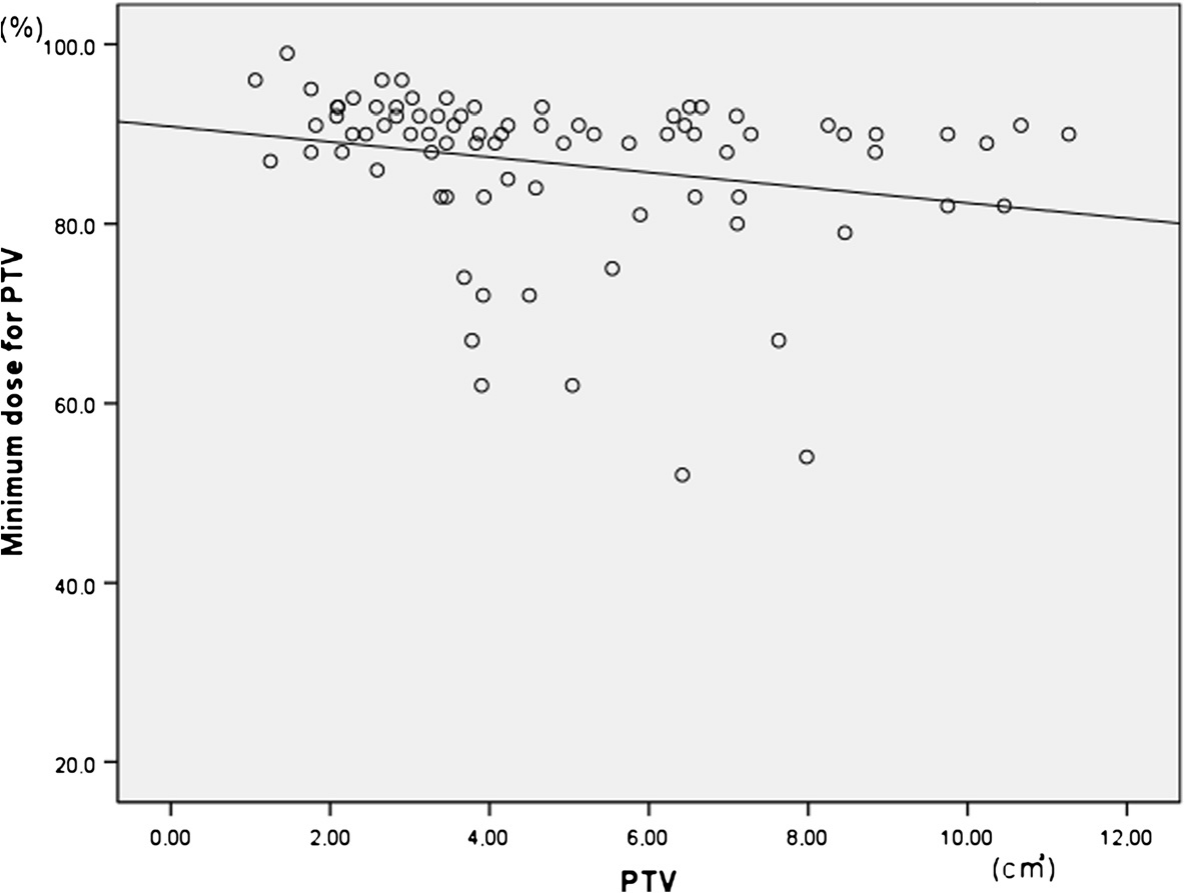
**Correlation of Minimum dose for PTV (%) and PTV.** Minimum dose for PTV (%) tended to be slightly lower for larger PTV.

This study also showed that T factor is the most significant factor affecting local control after stereotactic radiotherapy for stage I NSCLC. Several studies have shown that local recurrence is more frequent for larger GTVs [2,3,15] and that tumor size is an independent prognostic factor for stage I NSCLC patients [14]. A larger tumor tends to have hypoxic cells and cancer stem cells, which are more aggressive and resistant to radiotherapy [16], and a larger tumor would therefore have more time to metastasize than a smaller one. Cancer stem cells are recognized as a source of local or distant relapse [17]. A tendency to decrease the dose in the tumor periphery would also exacerbate the situation. In this study, the minimum dose for PTV (%) tended to be slightly lower for larger PTV (Figure 4). In addition, 66.7% of the patients with a T2 tumor and 38% of the patients with a T1 tumor were prescribed 7.5 Gy/fraction ×8 fractions (105 Gy BED_10_) or 4 Gy/fraction ×15 fractions (84 Gy BED_10_). Hence, T2 tumors tended to be treated with longer overall radiation time than did T1 tumors.

Figure 1 shows that there was a significant difference between local control rates in patients with T1 and T2 tumor. There was a decline in local recurrence and a plateau at about three years with no local recurrence after that period, although late recurrences at 5 years or more have been reported [18]. Local control in our hospital was almost same as several reports (Table 4). The 3-year survival rate in our study was better than that in other studies. It might be due to good collaboration with other departments and/or other hospitals, that is, not only death of primary tumor but also death of other causes more fewer than previously, because three-year cause specific survival rate was 97.0 (%), and it was almost same as some reports [19]. Relatively good survival rate could also imply that additional treatments after relapse including chemotherapy, re-irradiation, and best supportive care can influence survival. In addition, operability and PS may have affected the results of survival.

**Table 4:** Reports of stereotactic radiotherapy for stage I non-small cell lung cancer

**First author (Reference No.)**	**Number of patients**	**Total dose (Gy)**	**Single dose (Gy/fraction)**	**BED**_**10**_**(Gy)**	**Median follow up (months)**	Local control (%)**	**3-year overall survival (%)**
Timmerman [6]	55	60	20	180	34.4	98.2	55.8
Nagata [5]*	42	48	12	105.6	30	97.8	83
Fakiris [7]	70	60, 66	20, 22	180 - 211.2	50.2	94.3	42.7
Onishi [3]*	257	18 - 75	4.4 - 35	57.6 - 180	38	88.7	56.8
Our results*	80	48 - 60	4 – 12	84 – 105.6	30.4	92.5	89.9

BED_10_ is biological effective dose (BED) calculated from prescribed dose.

* Dose is calculated at the iso-center.

**Local control (%) = (number of patients without local recurrence) / (number of all patients)×100.

### BED_10_

When two types of prescriptions, one with BED_10_ of 105.6 Gy and the other with BED_10_ of 105 Gy, were compared, local control rates differed markedly despite only a slight difference in BED_10_ (Figure 2).

BED calculated by the linear quadratic formula with no correction was used in this study. The factor of overall radiation time was therefore not taken into account. During prolonged radiation delivery, sublethal damage repair takes place, leading to a decreased effect of radiation [20]. Actually, there was no local recurrence in patients with the shortest radiotherapy (105.6 Gy BED_10_) in this study, although 6 patients treated with 105.6 Gy BED_10_ had a T2 tumor.

Many clinicians often use the linear-quadratic (LQ) model and BED to estimate the effects of various radiation schedules, but it has been suggested that the LQ model is not applicable to stereotactic radiotherapy because of its high dose per fraction [20]. By contrast, Fowler *et al*. reported that the LQ model fitted the radiation response of epithelial tissues < 23 Gy/fraction [16]. The best-fit model for tumor responses to stereotactic radiotherapy warrants further research.

Furthermore, we speculate that restriction of prescribed doses due to the vicinity of central structures and/or the radiation oncologist’s discretion in consideration of factors including performance status affected the control rate, resulting in this difference (Figure 2).

Some reports have shown that local control rate with over 100 Gy BED_10_ was higher than that with less than 100 Gy BED_10_[3], although a meta-analysis conducted by Zhang *et al*. showed no significant difference between BED_10_ < 100 Gy and BED_10_ ≥ 100 Gy [21]. Medium BED or medium to high BED were recommended by Zhang *et al*. In our study, 4 Gy/fraction ×15 fractions (84 Gy BED_10_) was suggested to be insufficient for treatment of stage I NSCLC, particular in patients with a T2 tumor.

Further studies are needed to clarify the optimal total dose and fractions and the risk factors for relapse and side effects. When lower BED has to be prescribed because of tumor size, location of the tumor, and/or complications, use with chemotherapy can be considered [22].

### Minimum dose for PTV (%)

Wald χ^2^ test indicated that minimum dose for PTV (%) was the strongest prognostic factor. We carried out the calculation of the local control, grouping by the minimum dose for PTV (%) into 2 groups: the minimum dose for PTV (%) ≥ 89.88% and < 89.88%, and there was significant difference. Baumann et al. showed by univariate analysis that radiation dose calculated in equivalent doses in 2 Gy fractions (EQD2) at the periphery of the PTV had an impact on survival but not on local recurrence rate [15]. A trend toward smaller PTV margins for recurrent patients was also observed in their phase II trial [23]. Among multiple factors, BED at the PTV margin was found to be the only significant factor influencing local control by Wulf et al. [24].

BED at the PTV margin can decrease for many reasons. The internal margin is not constant in each fraction, and sufficient management of the respiration factor is therefore important for planning particularly in the lower lobe. Dosimetric problems also arise from the limited accuracy of dose calculation algorithms in treatment planning systems. Lax et al. reported that the pencil beam algorithm significantly overestimates the dose [25]. Radiation oncologists must have knowledge of the characteristics of the algorithm used in each institution. According to some reports, irradiation for large volumes of the lung can result in high-grade radiation pneumonitis [26]. Radiation pneumonitis is the most important adverse reaction in stereotactic radiotherapy for a lung tumor and its prevention is a crucial task because of its severity. For this reason, the leaf margin might be reduced at the discretion of the radiation oncologist when the target is large and/or the patient has respiratory complications.

Efforts to avoid unnecessary reduction of the margin should be made. Nowadays, we use the dose prescribed by 95% of the PTV (D95) for the stereotactic radiotherapy. We will conduct further research to determine an acceptable minimum dose (%).

The continuing development of technologies including respiratory gated radiotherapy and real-time tumor tracking should enable further reduction of the margin for PTV. Therefore, further dose escalation for a larger tumor such as a T2 tumor may be possible, and severe side effects may be reduced since the amount of normal tissue irradiation will also be reduced with reduction of the margin for PTV.

## Conclusion

T factor, BED_10_, and minimum dose for PTV (%) influence the local control rate. Local control rates can be improved by securing the minimum dose for PTV when radiation oncologists produce a radiotherapy plan for stage I non-small cell lung cancer in stereotactic radiotherapy.

## Competing interests

The authors declare that they have no competing interests.

## Authors' contributions

KJ and MK conceived of the study. YS and MK participated in data collection. YS performed the statistical analysis and drafted the manuscript. KJ, MK, KT, and NK helped to draft the manuscript. KJ, TS, and HM critically reviewed the article. All authors read and approved the final manuscript.
